# A need to accelerate health research productivity in an African University: the case of Makerere University College of Health Sciences

**DOI:** 10.1186/s12961-017-0196-6

**Published:** 2017-04-21

**Authors:** Damalie Nakanjako, Dickens Akena, Dan K. Kaye, James Tumwine, Elialilia Okello, Annettee Nakimuli, Andrew Kambugu, Hazel McCullough, Harriet Mayanja-Kizza, Moses R. Kamya, Nelson K. Sewankambo

**Affiliations:** 1 Department of Internal Medicine, Makerere University College of Health Sciences, P.O. Box 7072, Kampala, Uganda; 2 Department of Psychiatry, Makerere University College of Health Sciences, Kampala, Uganda; 3 Department of Obstetrics and Gynaecology, Makerere University College of Health Sciences, Kampala, Uganda; 4 Department of Paediatrics, Makerere University College of Health Sciences, Kampala, Uganda; 5 Infectious Diseases Institute, Makerere University College of Health Sciences, Kampala, Uganda; 6 London School of Hygiene and Tropical Medicine, London, United Kingdom

## Abstract

**Background:**

In the last decade, Makerere University College of Health Sciences (MakCHS) has taken strides in research and training to improve healthcare through collaborative training and research programs. However, there is limited data on the trends of MakCHS faculty contributions to research and on faculty growth to take leading roles in health research. This paper reviews MakCHS faculty research publications over 15.5 years and outlines possible strategies to enhance faculty research outputs.

**Methods:**

We used a mixed methods approach. A systematic review of research publications by faculty at MakCHS (PubMed and Google Scholar from January 1, 2000, to June 30, 2015) to quantify the number of research articles, areas researched, authorship contribution by MakCHS faculty, source of funding, as well as affiliated local and international collaborations. Graphs were used to shown trends in publications and leadership of authorship by faculty. Annual individual faculty research productivity was presented as publication per capita. Qualitative data on high priority needs to improve research outputs was collected through focus group discussions (FGDs) with faculty members, and analysed manually into emerging themes.

**Results:**

Of 298 faculty at MakCHS at 2015, 89 (30%) were female and 229 (77%) were junior and mid-level faculty (senior lecturer and below). The PubMed and Google Scholar searches yielded 6927 published articles, of which 3399 (49%) full-text articles were downloaded for analysis, 426/3825 (11%) available as titles/abstracts only, and 598/4423 (14%) were excluded. Only 614 articles were published in 2014, giving a publication per capita of 2.1 for any authorship, and 0.3 for first and last authorship positions. MakCHS faculty increasingly contributed as first, second, third, and last authors. Up to 57% of research was in infectious diseases, followed by non-communicable diseases (20%) and non-communicable maternal child health (11%). Priority needs to improve research outputs, as expressed by faculty, were (1) an institutionally led faculty career development program, (2) skills building in research methods and scientific writing, (3) protected time for research related activities, (4) opportunities for collaborative research, and (5) use of individual development plans.

**Conclusion:**

Faculty research productivity was low and dominated by infectious diseases and non-communicable disease research. There is a need for structured institutional support to optimise faculty research outputs. Only with increased research productivity will MakCHS and other academic institutions be able to make a significant contribution in addressing national health challenges.

**Electronic supplementary material:**

The online version of this article (doi:10.1186/s12961-017-0196-6) contains supplementary material, which is available to authorized users.

## Background

Research at academic institutions is relevant to influence national health policies to improve service delivery and health outcomes [1, 2]. Between 2000 and 2015, Makerere University College of Health Sciences (MakCHS) took huge strides in research and training to improve healthcare through collaborative training programs at undergraduate, masters and doctoral levels, in addition to non-degree skills training courses for African scientists [3–5]. These efforts have increased the pool of upcoming clinician scientists in the fields of HIV/AIDS and related illnesses, malaria and tuberculosis, which collectively carry the largest burden of illnesses in the sub-Saharan Africa region [3, 6–8]. However, there is limited data on trends of contributions by MakCHS academic faculty to research, relevance of academic research to local healthcare needs, and faculty growth to take leading roles in health research.

In addition to the social responsibility to address global health challenges [9], academic faculty participation in research is essential to optimise individual and institutional advancement, as well as faculty productivity, satisfaction and retention [10, 11]. With the ever increasing competitive nature of research funding, both institutional and individual track records in research productivity are strong contributors to career growth and institutional ranking [11, 12]. Therefore, monitoring and evaluation of faculty research productivity could motivate institutional leaders to nurture a culture of developing prolific publishing [13], in addition to high quality pedagogical skills.

We aimed at generating evidence on MakCHS faculty engagement in research over a period of 15.5 years by documenting the areas researched, levels of authorship contribution and source of funding, as well as affiliated local and international collaborations. This work builds on a previous report of 4-year data that 58% of research publications between 2005 and 2009 were led by MakCHS faculty or students as first authors [2]. Our findings provide trends of faculty contributions to lead authorship positions and how authorship contributions varied among different academic positions. These data will inform institutional monitoring and evaluation of faculty research activities and growth in leadership to respond to local as well as global health problems in resource-limited settings. Knowledge of the most researched areas, collaborations and multidisciplinary nature of research activities will inform the establishment/strengthening of thematic areas in line with the prevailing healthcare needs. We also determined high priority faculty needs that, if addressed, would enhance faculty research outputs. Data on faculty needs in terms of support for research will inform the institutional strategic plan to optimise faculty engagement in research and research outputs. We anticipate that documentation and regular feedback on on-going research and faculty engagement status would motivate individual faculty, as well as institutional, governmental and foreign agencies to support research relevant to prevailing health needs.

## Methods

### Study setting

MakCHS is the medical school at Uganda’s oldest and largest public university (founded in 1922). MakCHS initially started as a Faculty of Medicine in 1924 until 2008, when it was reconstituted as a college consisting of four schools, namely Makerere University School of Biomedical Sciences, Makerere University School of Health Sciences, Makerere University School of Medicine, and Makerere University School of Public Health. The college is headed by a principal and each school is headed by a Dean. MakCHS’ mission is to improve health and promote health equity by providing quality education, research and health services. MakCHS offers 12 undergraduate programs and 31 graduate programs.

### Data collection

We used a mixed methods approach comprising of firstly, a review of research publications by faculty at MakCHS to quantify the number of research articles, areas researched, authorship contribution, source of funding, as well as affiliated local and international collaborations, from January 1, 2000, to June 30, 2015. Secondly, qualitative data was collected from focus group discussions (FGDs) with faculty members at departmental level to determine high priority needs to improve faculty research outputs. This work was approved by the School of Medicine Research and Ethics Committee, and written informed consent was obtained from the faculty members that participated in FGDs.

### Search strategy

We conducted a review of published work by MakCHS faculty through PubMed and google scholar searches by last name of faculty, as listed on the human resources list and MakCHS departments. The human resources list included first, last, and middle (where applicable) names of faculty, current academic position, department of service, and employment status (permanent, contract or honorary). The search was filtered by publication date from January 1, 2000, to June 30, 2015, and the search words are shown in Additional file 1. Two independent searches were conducted by two faculty career development working group members (Nakanjako and Akena), who screened articles for inclusion and exclusion from analysis. Articles were included if they had at least one MakCHS faculty listed as an author or in acknowledgements to reflect all contributions made by faculty. Duplicates (appearing in both PubMed and Google Scholar) and articles completely irrelevant to health were excluded. Full-text research articles were downloaded and extracted into an excel sheet for analysis using a data extraction form with variables to assess gender of MakCHS faculty author, rank and department of the publishing faculty, authorship position, subject area of research, collaborations involved, and funders of the research. Titles/abstract only articles were excluded, if they had insufficient data to complete the required fields. If a publication had authors from more than one department, it was counted under each of the departments and faculty contribution to authorship was assigned to each author listed, although it was counted as one record under analysis of MakCHS publications. Data was analysed using frequencies and proportions to show the subject areas of research, collaborations, source of funding, and contributions by faculty in the various academic positions at MakCHS. Graphs were used to show trends in authorship leadership by faculty. Individual faculty research productivity was presented as publication per capita; calculated by the number of publications in a year, divided by the number of faculty in post during the same year. For this analysis, publication per capita was calculated for 2014, the most recent year in the study period and the year with complete human resources records of employees in post for the entire year.

Using a guide, FGDs were held at departmental level to document individual and institutional needs to enhance engagement of faculty in research-relevant activities. A convenient sample of two surgical departments (Obstetrics and Gynaecology, Anaesthesia), and two medical departments (Internal Medicine, Paediatrics) in the School of Medicine, that had at least nine faculty members to participate in FGDs, was used. In addition, two FGDs that included members from all departments were conducted. FGDs were conducted until saturation of ideas. A total of six FGDs were conducted by faculty career development group members. Each FGD consisted of at least nine faculty members and lasted 1–2 hours. Faculty were asked, ‘In your opinion, what are the major challenges that limit your research productivity?’, ‘What support do you need to improve your research productivity and career development?’, ‘How could your department support you to improve your research productivity?’, and ‘How could MakCHS support you to improve your research productivity and career progression?’. Data from FGDs were recorded (text and audio), transcribed and analysed manually according to emerging themes to prioritise faculty needs to improve individual and institutional research outputs.

## Results

### Faculty engagement in research

**Fig. 1:**
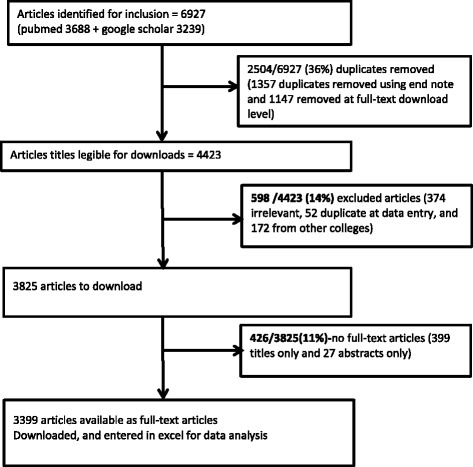
Flow chart showing the articles published by faculty at Makerere University College of Health Sciences between 2000 and 2015

Of 298 faculty at MakCHS in 2015, 89 (30%) were female and 229 (77%) were junior and mid-level faculty at levels of senior lecturer and below (Table 1). Overall, the PubMed and Google Scholar search yielded 6927 published articles, of which 3399 (49%) full-text articles were downloaded for analysis, excluding duplicates (articles that appeared in both the PubMed and Google Scholar outputs) and 426/3825 (11%) that appeared as titles/abstracts only without sufficient data to complete the required variables (Fig. 1). Of the 3399 publications in 15.5 years, 614 were published in 2014, of which 104 (17%) had MakCHS faculty as first author and 96 (16%) had a MakCHS faculty as a last author. The publication per capita for 2014 was 2.1 (considering the 298 faculty in post) for any authorship position, and 0.3 when only first and last authorship positions were considered.

**Table 1:** Description of academic faculty and research at Makerere University College of Health Sciences

Faculty (298)	*N* (%)
Description if academic faculty	
Sex	
Female	89 (30)
Faculty position	
Senior faculty (Assoc. Prof & Prof)	49 (16)
Junior and mid-level faculty (senior lecturer & below)	229 (77)
Honorary faculty	20 (7)
Faculty members in schools	
Medicine	159 (53)
Public health	54 (18)
Biomedical sciences	51 (17)
Health sciences	34 (11)
Description of published research	
Area of research	
Infectious diseases	57%
Non-communicable diseases	20%
Maternal child health (non-communicable)	11%
Health systems	8%
Education/capacity building	4%
Others	1%
Emerging/re-emerging diseases	0%
Research setting	
Hospital-based studies and cohorts	60%
Community-based Other	40%

#### Research areas

**Fig. 2:**
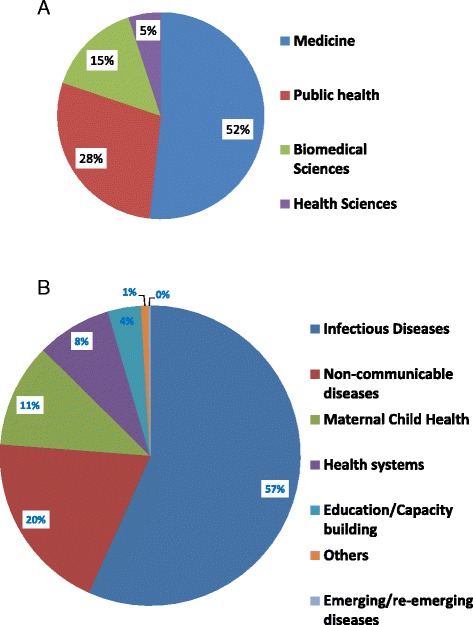
Research areas addressed by research publications by Makerere University College of Health Sciences (MakCHS) faculty between 2000 and 2015. **a** Contribution by the four schools at MakCHS. **b** Leading research areas

Over half (53%) of the research publications were by faculty from the School of Medicine, followed by School of Public Health (28%) and School of Biomedical Sciences (17%). Up to 57% of research was in infectious diseases, followed by non-communicable diseases (NCDs) at 20% and non-communicable maternal child health illnesses at 11% (Fig. 2). The majority (60%) of the research was hospital based, while 40% was based on studies in the community (Table 1).

#### Faculty contribution to research publications

**Fig. 3:**
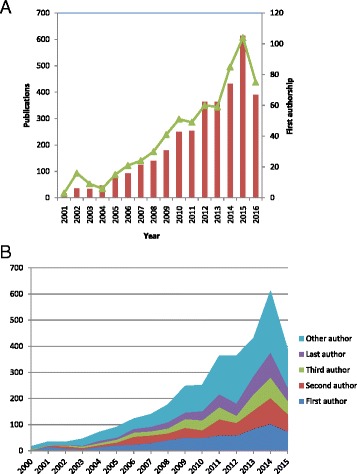
Authorship contribution of Makerere University College of Health Sciences faculty to published research in the last 15 years. **a** Number of faculty that contributed as first authors. **b** Number of faculty that contributed in various authorship positions between 2000 and 2015

**Fig. 4:**
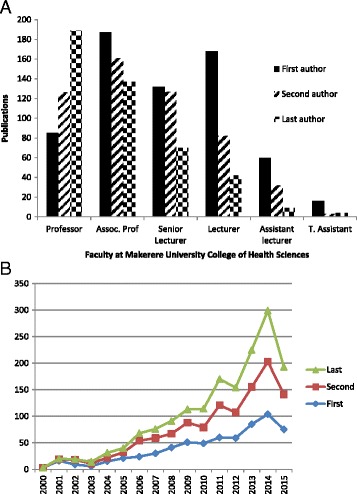
Leadership of authorship by faculty at Makerere University College of Health Sciences between 2000 and 2015. **a** Contribution to first, second and last authorship at all positions held by faculty. **b** Contribution to first, second and last authorship by senior faculty (Associate Professors and Professors)

In general, there was an increasing trend of MakCHS faculty contribution as first, second, third and last authors (Fig. 3). Senior faculty (Professor and Associate Professor) were taking leading roles as last authors and first authors. Junior faculty and mid-level (senior lecturer and below), were also increasingly taking on leading roles as first authors (Fig. 4).

#### Funding and collaborating institutions

Only 2172/3399 (64%) publications had information on research funding, of which 883/2172 (41%) were funded by institutions in the United States and Canada, 491/2172 (23%) were funded by European institutions and 133/2172 (6%) were locally funded in Uganda.

Up to 2278/3399 (67%) publications had data on collaborations, of which 1010/2278 (44%) involved collaborations with in-country institutions, 539/2278 (24%) had collaborations with academic institutions in the United States and 322/2278 (14%) had collaborations with institutions in Europe (Table 2).

**Table 2:** Sources of funding and collaborations for published research at Makerere University College of Heath Sciences between 2000 and 2015

Sources of funding (*n* = 2172)^a^	*N* (%)
National Institutes of Health/Fogarty	608 (28)
Europe – Sida, SWISS	491 (23)
Other United States/Canadian funding agencies	275 (13)
United Kingdom – Wellcome Trust, DFID	155 (7)
Uganda	133 (6)
Africa	34 (1.5)
China and other Asian countries	12 (0.5)
None/not listed	464 (21)
Collaborations (*n* = 2278)^b^	
Uganda-based academic institutions/partners	1010 (44)
United States academic institutions/partners	539 (24)
African academic institutions/partners	154 (7)
Europe academic institutions/partners	322 (14)
United Kingdom academic institutions/partners	101 (5)
Canada-based academic institutions/partners	33 (1)
Others – India, China	20 (1)
None reported	99 (4)

^a^1236 – funding data missing, ^b^1124 – data on collaborations missing

#### Faculty needs to improve research outputs

Overall, six FGDs were conducted with a total of 72 staff participating, of whom 57 were junior and mid-level faculty (19 (33%) female) and 15 were senior faculty (7 (47%) female). Below are the needs that academic faculty felt should be addressed to improve their research outputs (Table 3).

**Table 3:** Priority areas to improve faculty engagement in research as highlighted by Makerere University College of Heath Sciences faculty

Themes	High priority needs as expressed by faculty
Institutional support for faculty growth in research	Need for a structured implementation of faculty career development functions including monitoring and evaluation
	Skills’ building in research methods and scientific writing
	Support with manuscript writing to publish dissertations and abstracts in peer-reviewed journals
	Protected time for research related activities
Opportunities for collaboration	Limited opportunities to engage in collaborative research
	Need for multidisciplinary research interest groups
Individual development planning	Balancing research, clinical care and administrative responsibilities
	Time management
	Use of individual development planning
	Balancing career and family needs

1. **Need for an institutionally led structured faculty career development program**: “*A faculty career development program is long overdue. There are people within our institution that were appointed and reached retirement at the same rank. This is unfair!*” said one senior faculty. “*It is assumed that our institution supports staff career development but it is unstructured and many of us have not read our human resource manual*.” “*We are always talking about mentoring our students and we forget that we also need to be mentored*”, said one junior faculty.
2. **Skills’ building in research methods and scientific writing:** “*We need regular workshops to improve individual skills in research, teaching and assessment methods, and time management*”, said one junior researcher. “*Many of us have dissertations that are not yet published. We need support to publish the dissertations in peer reviewed journals*”, said one head of department. “*We need statistical support to analyse data and write up abstracts and reports that we present at local and international meetings*”, said one lecturer.
3. **Protected time for research related activities:** “*I need to travel abroad, away from the heavy clinical schedules, to complete my manuscripts*”, said one junior faculty*.* “*We spend unlimited time on wards and in theatres, leaving us with limited time to develop research proposals*”, said another faculty. “*All masters’ dissertations are lying on the shelves. Publishing them requires time which the faculty do not have*”, said one head of department.
4. **Opportunities for collaborative research:** “*We miss out on many of the research grants advertised that require international collaborators. How do we get the international collaborators?*” asked one senior faculty. “*How do we use our own capacity to develop the capacity of others? We need research groups with a mix of senior and junior faculty to allow senior people to mentor younger faculty in research*”, one senior researcher explained. “*It has been difficult for me to find a local mentor. I need to travel abroad to work with my mentor, which is expensive*” said one junior faculty.
5. **Individual development planning and time management:** Majority of the faculty admitted that they had never written personal career development plans. “*What is the average period a faculty should stay at one academic position before promotion*?” one junior faculty asked. “*I work in private clinics on locum to meet my family financial needs. This leaves me with no time for research and writing*”, said one junior faculty.

## Discussion

### Faculty growth and contribution to research publications

We found a rising rate of dissemination of research through peer-reviewed publications within each cadre of academic faculty. There was a trend of increasing first author contributions particularly among lecturers and associate professors, as well as an increasing trend of last author contributions among Professors and Associate Professors. A majority of faculty were participating in research as co-authors in positions other than first, second, third or last positions. These data reflect growth in academic research leadership among faculty at MakCHS during the study period of 15.5 years. These results are comparable with data from a survey of over 3000 western academic institutions, where research publications increased with faculty rank [12, 13]. We reported low research productivity as measured by a publication per capita of 2.0 in 2014 for any authorship position, and 0.3 for first and last author positions. We were unable to determine the annual publication per capita because we did not collect data of faculty in post annually. Longitudinal data on annual staff recruitment and promotion is required to enable monitoring and evaluation of trends in publication per capita as one of the measures of institutional research productivity. Our data is comparable with data from family medicine schools in America and the American University of Beirut, where faculty produced 1–2 scholarly products a year since they had clinical and academic demands with limited time allocation for research [14, 15]. We recommend development of institutional tools to measure and monitor research grants, publications and supervised doctoral students, which have previously been used to determine research output scores among faculty in academic institutions in America [16].

### Research focus and relevance

Infectious diseases dominated the focus of research at MakCHS, reflecting the burden of disease in the region. In the 2010 and 2012 WHO reviews of global burden of diseases [6, 7], HIV/AIDS, tuberculosis, malaria, lower respiratory infections, and meningitis were the leading causes of mortality in Uganda, hence the relevance of infectious diseases research at MakCHS to meet the diseases of highest burden in the country. NCDs (including cardiovascular diseases, cancers, chronic pulmonary diseases, diabetes, mental illness), and non-communicable maternal and child health illnesses emerged as the second and third most researched areas, respectively. This trend is consistent with available evidence of increased global burden of NCDs, with low- and middle-income countries contributing 80% of NCD-related deaths [17, 18]. WHO estimates show that, by 2030, 80% of deaths globally will be attributable to NCDs [19, 20]. Among young populations aged 35 years and under in sub-Saharan Africa, the prevalence of hypertension ranged from 9% in an Ethiopian population, 27% in a Ugandan rural adult community [21], to 48% in a Mozambican population, and reaching 70% in an elderly (≥ 70 years) urban Tanzanian population [22]. Therefore, research led by MakCHS faculty is well positioned to meet the emerging burden of NCD-related premature deaths and disability in the region.

However, we found limited research on emerging and re-emerging diseases, including Ebola and other haemorrhagic fevers, which have caused life-threatening epidemics in the sub-Saharan Africa region in the past decade [23]. Given the experience Uganda has had with haemorrhagic fever epidemics [24, 25], opportunities exist for academic faculty at MakCHS to contribute to the development of novel interventions towards surveillance and management of Ebola [26]. We propose a strategic review of local human resource, research funding and infrastructure to improve surveillance and preparedness of health systems to handle these epidemics. Similarly, research was dominated by hospital-based, followed by community-based studies. There was limited pre-clinical basic science research, reflecting the challenge of limited infrastructure, human resource and funding in this area [27]. There is clearly a need for strategies to revamp the institutional infrastructure for pre-clinical research to nurture innovations to improve clinical care. We postulate that increasing exposure to basic science research earlier on in medical training will inadvertently increase the use of basic science research tools to answer relevant clinical questions.

### Faculty needs to optimise research outputs

Overall, faculty needed (1) an institutionally led structured career development program; (2) skills’ building in research methods and scientific writing; (3) protected time for research related activities; (4) opportunities for collaborative research; and (5) individual development planning and time management. Our results are comparable with reports from medical school faculty in America, where up to 42% seriously considered quitting academic careers because of absence of faculty development programs, difficulties balancing work and family, lack of recognition of clinical and teaching commitments, and lack of regular evaluation of academic progress [28]. A further understanding of unique enablers and hindrances to research productivity in the different schools is required to optimise faculty production, satisfaction and retention. The listed needs were subsequently used to develop a college career development program that aims to support academic career progression in research, teaching, healthcare delivery and resource mobilisation. Faculty mentoring greatly influences personal development and research productivity, including publication and grant success [29, 30]; however, implementation research is needed to guide evidence-based interventions to address the identified gaps in institutional support of faculty productivity.

We found that 426/3825 (11%) of faculty research appeared as titles/abstracts only without full publications in peer-reviewed journals. Qualitative data from faculty also revealed that a bulk of research theses by faculty and graduate students continue to lie on unit shelves without publication in peer-reviewed journals. Part of the reasons highlighted included limited skills, mentorship and time for productive scientific writing, as expressed by faculty. Unpublished research theses and reports are low hanging fruits that could be targeted to increase faculty and institutional research publications. Strategies to bridge the do–publish gap of research at MakCHS are clearly needed to minimise the research–policy–practice gaps in healthcare. Our results also emphasise individual and institutional challenges in developing functional and multidisciplinary research groups, grant writing skills and networks between local and international scientists in relevant fields [5]. These gaps present untapped opportunities for institutional support to enhance faculty productivity.

### Funding for research

Noteworthy is the fact that a majority of published research was funded by foreign agencies, with the National Institutes of Health and European agencies as the leading funders. Indeed, there has been an increase in funding for research in Africa in the last decade [31], and institutions need to prepare researchers to apply for these opportunities. Therefore, mentoring MakCHS faculty in competitive resource mobilisation for research is critical for sustainability of their engagement in research. Similarly, there is need for heightened advocacy for local research funds to answer locally relevant questions in disease prevention, diagnosis, treatment and surveillance. There is need for academic institutions to re-emphasise that new national investment in health research is required to optimise the utilisation of clinical-scientific discoveries to improve patient care [32]. We also noted that data on author affiliation, collaborations and source of funding was incomplete or missing in 20%, 33% and 36% of full-text publications, respectively, hence our recommendation of institutional emphasis on complete documentation of these areas, which remain necessary for faculty research track record as well as institutional credibility and ranking [33].

MakCHS continues to seize opportunities for faculty to actively participate in research through research-and-training partnerships between universities in high- and low-income countries [5] such as the Swedish International Development Cooperation Agency (Sida), the Medical Education Partnership Initiative (MEPI)’s Medical Education for Equitable Services to all Ugandans (MESAU), Supporting Policy Education for policy Evidence-based Decisions (SPEED), Training Health Researchers into Vocational Excellence in East Africa (THRiVE), Makerere University/Uganda Virus Research Institute Infection and Immunity program (MUII), The Netherlands Organization for International Cooperation (NUFFIC), Norwegian Agency for Development Cooperation (NORAD), and the Resilient Africa Network (RAN), among others [3, 5]. In a review of research outputs from 12,400 Norwegian university researchers, availability of research funds, teaching loads, workload policies, departmental culture and organisational context were critical for an institutional environment to optimise productivity of its talented and hardworking faculty [34]. Institutional academic research expenditures, number of post doctorates and number of doctoral recipients/candidates accounted for over 90% of the variability in publication outputs in the Norwegian science and engineering universities [35]. We, however, did not evaluate instructional and student learning productivity, both of which are key elements of faculty performance [13]. We recommend comprehensive specific monitoring and evaluation programs for faculty productivity and career growth including, among others, number of graduate programs, ranking of publications, citation index, publications:grants ratio, total funding acquired per faculty, and institutional awards or recognitions established for highly productive faculty.

## Conclusion

Faculty research productivity was low and dominated by infectious diseases and non-communicable disease research. There is a need for structured institutional support to optimise faculty research outputs. Only with increased research productivity will MakCHS and other academic institutions be able to make a significant contribution in addressing national health challenges. We recommend longitudinal monitoring and evaluation of individual faculty, and institutional research and instructional productivity.

## Additional file

Additional file 1: Search words. (DOCX 13 kb)
