# Supplementary material for: A need to accelerate health research productivity in an African University: the case of Makerere University College of Health Sciences
Source: Health Res Policy Syst. 2017 Apr 21;15:33. doi: 10.1186/s12961-017-0196-6 (PMC5399829; doi:10.1186/s12961-017-0196-6)
Supplement: Additional file 1: — Search words. (DOCX 13 kb) [file 12961_2017_196_MOESM1_ESM.docx]

**Search strategy:** We conducted a review of published work by MakCHS faculty through PubMed and google scholar searches by last name of faculty, as listed on the human resource (HR) list and MakCHS departments. The HR list included first, last, middle (where applicable) names of faculty, current academic position, department of service and employment status (permanent, contract or honorary). **The search words were** “Makerere University”, followed by “Medical School” OR “Faculty of Medicine” OR “College of Health sciences” OR “School of Biomedical sciences” OR “School of Bio-Medical Sciences” OR “School of Medicine” OR “School of Health Sciences” OR “School of Public Health” OR “Institute of Public Health” OR “Infectious Diseases Institute” OR “Department of Internal Medicine” OR “Department of Surgery” OR “Department of Obstetrics and Gynaecology” OR “Department of Obstetrics and Gynecology” OR “Department of Psychiatry” OR “Department of Family Medicine” OR, “Department of Anaesthesia” OR “Department of Anesthesia” OR “Department of Ear Nose Throat” OR “Department of Ophthalmology” OR “Department of Orthopaedics” OR “Department of Orthopedics” OR “Department of Radiology and Radiotherapy” OR “Medical Research Centre” OR “Reproductive Health Unit” OR “Department of Paediatrics and Child Health” OR “Department of Pediatrics and Child Health” OR “Department of Health Policy and Management” OR “Department of Epidemic and Biostatistics” OR “Department of Community Health and Behavioral Sciences” OR “Department of Disease Control and Environmental Health” OR “Department of Human Anatomy” OR “Department of Anatomy” OR “Department of Biochemistry” OR “Department of Microbiology” OR “Department of Pathology” OR “Department of Physiology” OR “Department of Pharmacology and Therapeutics” OR OR “Department of Medical Illustration” OR “Department of Pharmacy” OR “Department of Dentistry” OR “Department of Nursing” OR “Department of Allied Health Sciences” “Child health and development center”, clinical epidemiology unit”. These were filtered by publication date from 2000/01/01 to 2015/06/30. Two independent searches were conducted by two faculty career development working group members (Nakanjako and Akena), who screened articles for inclusion and exclusion from analysis. Articles were included if they had at least one MakCHS faculty listed as an author or in acknowledgements to reflect all contributions made by faculty. Duplicates (appearing in both Pubmed and google scholar) and articles completely irrelevant to health were excluded.
